# A Comparative Evaluation of the Mechanical Properties of Two Calcium Phosphate/Collagen Composite Materials and Their Osteogenic Effects on Adipose-Derived Stem Cells

**DOI:** 10.1155/2016/6409546

**Published:** 2016-04-28

**Authors:** Qing Li, Tong Wang, Gui-feng Zhang, Xin Yu, Jing Zhang, Gang Zhou, Zhi-hui Tang

**Affiliations:** ^1^National Engineering Laboratory for Digital and Material Technology of Stomatology, Beijing 100081, China; ^2^Center of Digital Dentistry, Peking University School and Hospital of Stomatology, Beijing 100081, China; ^3^2nd Dental Center, Peking University School and Hospital of Stomatology, Beijing 100101, China; ^4^Institute of Process Engineering, Chinese Academy of Sciences, Beijing 100190, China; ^5^Key Laboratory for Biomechanics and Mechanobiology of Ministry of Education, School of Biological Science and Medical Engineering, Beihang University, Beijing 100191, China

## Abstract

Adipose-derived stem cells (ADSCs) are ideal seed cells for use in bone tissue engineering and they have many advantages over other stem cells. In this study, two kinds of calcium phosphate/collagen composite scaffolds were prepared and their effects on the proliferation and osteogenic differentiation of ADSCs were investigated. The hydroxyapatite/*β*-tricalcium phosphate (HA/*β*-TCP) composite scaffolds (HTPSs), which have an additional *β*-tricalcium phosphate, resulted in better proliferation of ADSCs and showed osteogenesis-promoting effects. Therefore, such composite scaffolds, in combination with ADSCs or on their own, would be promising for use in bone regeneration and potential clinical therapy for bone defects.

## 1. Introduction

Bone loss due to trauma, inflammation, and surgical processes has posed great difficulty in the aesthetic reconstruction of a functional alveolar bone [1, 2]. Tissue engineering and biomaterials, which can promote alveolar bone regeneration, have become a popular focus of current studies [3–6].

As an important element in alveolar bone tissue engineering, osteogenic cells, in addition to growth factors and scaffolds, have been studied extensively to regenerate new bones and to repair large bone defects. Embryonic, osteoblastic, and adult stem cells have been adopted for such applications [7]. Mesenchymal stem cells (MSCs) from the bone marrow stem cells (BMSCs) and from the adipose-derived stem cells (ADSCs) are two of the most extensively explored cell sources for bone regeneration [8]. However, the collection of the bone marrow is a painful operation and the yield of MSCs from this source is relatively low [9]. Adipose tissue is an abundant source of ADSCs and can be easily obtained by using liposuction, which is less traumatic than bone marrow collection [10]. ADSCs can undergo osteogenic differentiation in vitro under certain conditions [11, 12] and yield 100- to 500-fold more MSCs than BMSCs after initial harvest [13]. ADSCs preserve their proliferative and differentiation potentials even in older patients, which would be helpful for repairing bone defects in these patients [7]. In addition to these properties, the nonimmunogenic properties [14] and capacity to migrate to the injury site in ADSCs make them a potential candidate for use in alveolar bone regeneration [15, 16].

The materials used for the preparation of bone grafts must be biocompatible, osteoinductive, and osteoconductive. Additionally, the structure and mechanical properties of these materials should be similar to those of natural bones [17]. Among the different kinds of biomaterials, calcium phosphate-based materials including hydroxyapatite (HA), *α*-tricalcium phosphate (*α*-TCP), and *β*-tricalcium phosphate (*β*-TCP) have been widely explored for use as scaffolds in bone regeneration [18–20]. As a major mineral constituent of natural bones, HA can maintain space for the proliferation of osteogenic cells and for the exchange of substances due to long-term resorption, while TCP can form a porous structure and release calcium and phosphorus ions by dissolving, which contributes to osteogenic activity and thus to new bone formation [21, 22]. A composite material comprising HA and *β*-TCP was developed, which incorporated the advantages of both these materials, showing better mechanical properties and allowing better degradation [23, 24]. Recently, many researchers have attempted to bring about improvements in such composite scaffolds by modifying many parameters such as the HA/TCP ratio as well as phase composition, formulation, sizes, and shapes [25–27].

While extensive research has been conducted on the effects of HA and TCP on BMSCs [28–31], relatively fewer studies have revealed the influence of these biomaterials on the osteoblastic differentiation capacity of ADSCs. Hicok et al. showed that, in combination with ADSCs, HA and *β*-TCP could enable the formation of osteoids in mice with severe combined immunodeficiency (SCID) [32]. Moreover, Lee et al. revealed that HA could induce osteogenic differentiation [33]. Further, ADSCs seeded on HA-TCP scaffolds could form a higher percentage of osteoids in mice with SCID compared to that observed with the use of HA alone [32, 34]. Additionally, a *β*-TCP matrix could achieve similar effects when used with osteogenic culture media [32]. Based on these data, we hypothesized that a HA/*β*-TCP composite scaffold could better promote osteogenic differentiation than a scaffold comprising only either one of these biomaterials.

However, because of the brittleness of such inorganic materials, better effects could be achieved by additionally using organic constituents of natural bone, such as collagen [35]. Collagen, especially type-I collagen, which accounts for a major part of the extracellular matrix (ECM), has become one of the most widely explored tissue engineering scaffolds due to its excellent biocompatibility, degradation properties, and absorbability [36]. Collagen-based materials result in excellent osteogenic differentiation when used with different stem cells [37, 38]. Besides, in collagen-calcium phosphate composite materials, the poor mechanical properties of both the organic and the inorganic components, such as stability [39] and the mechanical “wet” properties [40], can be strengthened partly owing to the energy dissipation that occurs through a multiplicity of “sacrificial bonds,” which are covalent bonds formed within the collagen molecular chain itself or due to the crosslinking between different chains in the composite materials [41, 42]. In addition, ions or hydrogen bonds [43], the sliding of layered water films [44], and other hypothetical mechanisms of mineral-collagen interactions have been proposed to explain the improved nature of the composite materials. A combination of the adhesion property of collagen and the osteoconductive ability of HA and TCP can be used in such composite biomaterials to increase the proliferation and osteogenic differentiation of ADSCs [32, 43, 44].

In this study, we prepared pure HA particles (HAP) using the traditional solution precipitation method and HA/*β*-TCP particles (HTP) by burning the bovine bone and composited both these particles with type-I collagen by freeze-drying. In addition to the composition, microstructure, and mechanical and wet properties of these composite biomaterials, their effects on the proliferation and osteogenic differentiation potential of ADSCs were examined to pave the way for further application of ADSCs as seed cells to improve bone regeneration.

## 2. Materials and Methods

### 2.1. Materials

CaO (analytical reagent, AR), H_3_PO_4_ (AR), H_2_O_2_ (AR), NaOH (AR), and absolute alcohol (AR) were all purchased from Beijing Chemical Works (Beijing, China); type-I purity collagen was purchased from Collagen Biotechnology Co. Ltd. (Hebei, China); KBr (spectroscopically pure) was purchased from Botianshengda Co. Ltd. (Tianjin, China); phosphate buffer solution (PBS), Dulbecco's Modified Eagle Medium (DMEM), and fetal bovine serum (FBS) were all purchased form Thermo Fisher Scientific (USA); MTT and DMSO were purchased from Sigma (USA); Trizol reagent was purchased from Invitrogen (USA); SYBR Green I and the PrimeScript*™* RT reagent Kit was purchased from TaKaRa (Japan); and FITC phalloidin was purchased from Alexis (USA).

### 2.2. Preparation of the Composite Scaffolds

#### 2.2.1. Preparation of HAP

HAP was synthesized using a reaction of Ca(OH)_2_ and H_3_PO_4_ in water, based on the method described by Antebi et al. [45]. Briefly, in a CO_2_-free atmosphere filled with Ar, 1 M of CaO was added to 500 mL distilled water, and 300 mL of a H_3_PO_4_ aquatic solution (2 mol/L) was slowly added (at about 0.5 mL/min) to the Ca(OH)_2_ slurry to obtain a final Ca/P molar ratio of 1.67. After boiling for 2 days, the precipitated solid was collected by centrifugation and washed with distilled water. The precipitate was then redispersed in boiled distilled water. These washing and boiling procedures were repeated until the pH of the supernatant was approximately 7. Then, the HA precipitate was collected by centrifugation, washed with acetone, and dried at 110°C. The HA crystals were then ground and filtrated through an 80-mesh screen.

#### 2.2.2. Preparation of HTP

HTP was prepared by calcining bovine cancellous bone [46], which was cut into cubes of 4 mm × 4 mm × 10 mm. After being incubated in 30% H_2_O_2_ solution for 30 min, the bone cubes were then stewed in a NaOH solution for 1 h to remove collagen and proteins. The bone cubes were then washed with water and immersed in 1% H_3_PO_4_ solution and heated at 125°C for 2 h. This was followed by washing with 100% alcohol to remove moisture, which had resulted in small cracks during the heat treatment. The bone cubes were then air-dried for 12 h and heated in a furnace (siliconit muffle furnace, Kwangsung Science Co., Korea) at a rate of 10°C/min up to 1000°C. This process was continued for 2 h. Finally, the bone cubes were ground and filtered through an 80-screen cloth to obtain HTP particles with a diameter of 0.25–1 mm.

#### 2.2.3. Preparation of the HAP- and HTP-Collagen Composite Scaffolds (HAPSs and HTPSs, Resp.)

The composite scaffolds were prepared using the freeze-drying approach [47]. Because an acidic solution, such as acetic acid, would cause tissue inflammation, pure water was used to dissolve the collagen. Pure type-I collagen (Collagen Biotechnology Co. Ltd., China) was dispersed in distilled water at a concentration of 4 g/100 mL. A homogenizer (IKA, Germany) was used to achieve full dispersion. After the collagen was mixed with water thoroughly, HAP and HTP were added into slurries with constant stirring at 45 rpm for 24 h. The concentration of the bone particles was 36 g/100 mL. After degassing, the slurry was injected into stainless steel molds with dimensions of 5 mm × 5 mm × 10 mm. In order to densify the material, it was subjected to a constant pressure of 105 kPa for 2 h. The molds were then exposed to a temperature of −10°C for 2 h to freeze, and the mixture was then finally freeze-dried in vacuum to obtain HAPS and HTPS. The scaffolds were then exposed to 25 kGy gamma radiation for sterilization at room temperature.

### 2.3. Characterization of HAPS and HTPS

#### 2.3.1. Morphology Analysis

The surface morphologies of HAPS and HTPS were examined by SEM using a Hitachi S-4800 SEM (Hitachi, Japan) with 15 kV accelerating voltage. Before the examination, both samples were heated at 40°C for 2 h to remove residual moisture and then underwent spray-gold.

#### 2.3.2. Microstructure Analysis

In order to study the microstructures of the two samples, their N_2_ adsorption isotherms were measured using an AUTOSORB-1 adsorption analyzer (Quantachrome, USA) at −196°C. The specimens were then dried at 200°C for 24 h in a N_2_ atmosphere to remove the water on the solid surface. The specimens were then exposed to a temperature of 25°C in vacuum to attain a residual pressure of 10^−4^ Pa. The multipoint BET method was used to measure the specific surface area (*S*_*A*_), pore volume (*V*_*p*_), and average pore radius (*P*_*R*_) of the two specimens [46]. Each group had five samples and the results were displayed in the form of mean ± standard deviation.

#### 2.3.3. EDX Analysis

An Energy Dispersive X-ray (EDX) spectrometer (Hitachi S-4800) connected to the SEM was used to determine the elemental combination of the composite material. The specimens did not undergo spray-gold before examination.

#### 2.3.4. FTIR Analysis

FTIR analysis using Shimadzu FTIR-8400S was conducted for the chemical analysis of HAPS and HTPS. The spectral range was set as 4000 to 400 cm^−1^ with a resolution of 4 cm^−1^ and scan time of 100. The samples were diluted to a concentration of 1% by mixing with KBr (infrared grade).

#### 2.3.5. XRD Analysis

X-ray diffraction spectroscopy (XRD) was conducted to examine the crystal phase composition of the samples. A 0.5 mm × 0.5 mm area of each sample was randomly selected and the XRD spectra were acquired at room temperature using an X-ray diffractor (D/max-II; RIGAKU, Japan) with Cu K*α* radiation. The range was 10–90° with a 0.2° step and 1 s/step scan speed (40 kV, 40 mA).

#### 2.3.6. Analysis of the Mechanical Properties of HAPS and HTPS

To evaluate the strength of HAPS and HTPS, Young's moduli of all the specimens were determined using a Universal Testing Machine (Instron 300DX, US). Additionally, the Bio-Oss Collagen Young's moduli were also calculated as a positive control. The dimensions of each specimen were measured using a vernier caliper. These scaffolds were then placed on plates and compressed uniformly. They were then subjected to a force at the rate of 1 mm/min exerted on the top of the scaffolds. The load was augmented until the scaffolds crushed. Force and displacement data were recorded in order to generate the stress-strain curves. By measuring the slope of the stress-strain curve in the elastic region, Young's modulus could be calculated. Each group had five samples and the results were displayed in the form of mean ± standard deviation.

#### 2.3.7. Water Absorption Rate Assay

The water absorption rates of HAPS and HTPS were also determined. Before the water was soaked, the specimens were weighed using an analytical balance (*M*_0_) round to 0.001 g. The specimens were then immersed in 0.01 mol/L PBS (pH 7.4) for 15 min to achieve complete water absorption. The surface was then dried using filter paper and the specimens were weighed using an analytical balance (*M*_1_) with the same accuracy. The water absorption rate was calculated using the formula (*M*_1_ − *M*_0_)/*M*_0_. Each measurement for both the groups was repeated three times.

### 2.4. Evaluation of the Biological Effect of HAPS and HTPS on ADSCs

#### 2.4.1. Cell Culture

ADSCs (Poietics Human Adipose-Derived Stem Cells) were purchased from ScienCell (ScienCell, USA) and cultured in 100 mm dishes with Dulbecco's Modified Eagle Medium (DMEM, GIBCO, USA) supplemented with 10% (v/v) fetal bovine serum (FBS, GIBCO, USA). The medium was replaced every other day and cells were expanded until passage 3. The cells were then digested using a trypsin-EDTA solution (Sigma, USA) and cell number was calculated using a blood counting chamber. The cell suspension was then centrifuged for 5 min at 1200 rpm at room temperature and resuspended in DMEM (10% FBS) to adjust the cell number to 1 × 10^7^/mL. After placing the scaffolds in a 24-well tissue culture plate (CORNING, USA), each well was seeded with 200 *μ*L of the cell suspension. After 1 h of cell adhesion at 37°C with 5% CO_2_, 700 *μ*L of DMEM (10% FBS) was added to each well and the medium was replaced every day.

#### 2.4.2. Cell Proliferating Assay

After culturing for 1, 3, 5, and 7 days, cell proliferation was determined using the MTT method. In brief, each well was aspirated and washed with PBS three times. Next, 700 *μ*L of DMEM (with 10% FBS) and 70 *μ*L of MTT (0.5 mg/mL of water) solution was added into each well and incubated at 37°C at 5% CO_2_ for 3 h in the dark. The medium was then aspirated and 700 *μ*L of DMSO was added into each well to dissolve the formazan. After shaking gently on a shaker in the dark for 15 min, the absorbance at 450 nm was measured using a microplate reader (biokinetics reader, Bio-Tek Instruments, USA). Cells grown on the culture plates only were used as the control group. The measurements were performed in triplicate for each group.

#### 2.4.3. Quantitative Real-Time Polymerase Chain Reaction (RT-qPCR) Assay

Total RNA was extracted from the ADSCs after 3 and 5 days of culture on HAPS and HTPS using Trizol reagent (Invitrogen, USA) by following the manufacturer's protocol. SYBR Green I (TaKaRa, Japan) was used for quantitative real-time polymerase chain reaction and the PrimeScript RT reagent Kit was used for reverse transcription. The target PCR primers for ALP, BMP-2, OCN, OPN, COL1A1, COL2A, and ACTIN were designed using the Basic Local Alignment Search Tool® (Table 1). For each data point, there were three replicates. From each sample, cDNA was synthesized from 1 *μ*g of total RNA using reverse transcriptase (TaKaRa). The qPCR mixture in each tube contained 10 *μ*L of 2x SYBR Green I qPCR Mix (TaKaRa), 250 nM of each primer, PCR grade water, and 20 *μ*g of cDNA. The PCR cycling program was set as follows: 95°C for 5 min initially, followed by 40 cycles at 95°C for 15 s, 58°C for 30 s, and 95°C for 15 s. The comparative Ct method (also known as the 2^−ΔΔCt^ method) was applied to analyze the dissociation curves.

#### 2.4.4. Immunofluorescence Assay

In order to observe the morphology of the cells after seeding them on the scaffolds, the immunofluorescence staining of the cytoskeleton of the ADSCs was conducted. After 24 h of incubation on both scaffolds, the cells were gently washed with 0.01 M PBS three times and then fixed in 4% glutaraldehyde PBS solution for 20 min at room temperature. The cells were then again washed with PBS three times, and 0.5% Triton X-100 PBS solution was applied on the cells for 5 min to increase cell membrane permeability. After washing again with PBS, the cells were incubated with FITC phalloidin (Alexis, USA) for 20 min in the dark at room temperature. The cells were then washed with PBS three more times and then incubated in DAPI (5 *μ*g/mL) in the dark at room temperature for 5 min to allow visualization of the nucleus. After washing with PBS again three times, the samples were mounted with 95% glycerin PBS solution. A confocal microscope (Olympus IX71, Fluoview, Japan) was used to obtain the images. An excitation filter at 490 nm and an emission filter at 530 nm were used to visualize FITC staining and an excitation filter at 340 nm and an emission filter at 488 nm were used for DAPI staining.

### 2.5. Statistical Analysis

The data are represented as the mean ± SD. In terms of statistical comparison, this study adopted the *t*-test or one-way ANOVA as well as the LSD test. ^*∗*^ *P* < 0.05 was considered to indicate statistical significance.

## 3. Results

### 3.1. SEM Observation

**Figure 1:**
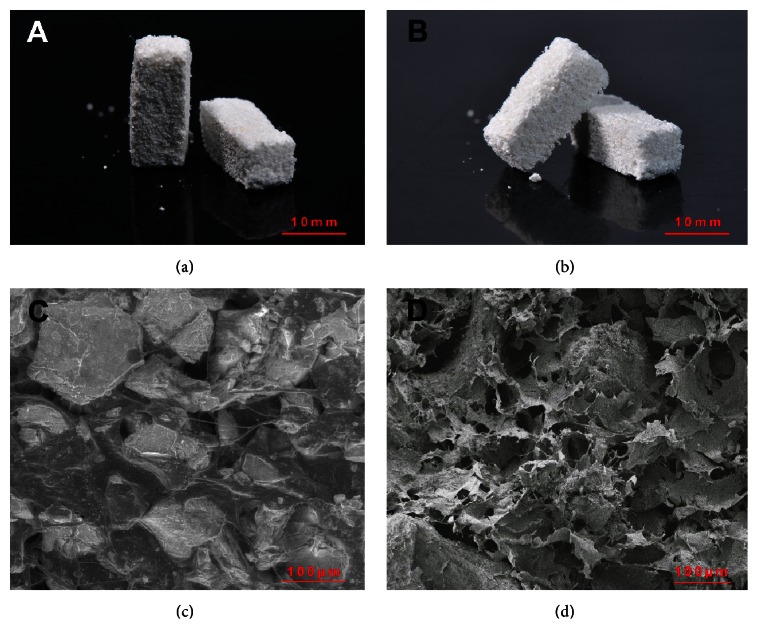
Pictures and SEM images of HTPS and HAPS. (a) HTPS group; (b) HAPS group; (c) SEM images of the HTPS group, 500x; and (d) SEM images of the HAPS group, 500x.

The pictures and SEM images of HAPS and HTPS are shown in Figure 1. The macroscopic shapes (Figures 1(a) and 1(b)) indicate that HAP and HTP were distributed in the collagen uniformly. The SEM images (Figures 1(c) and 1(d)) show that both the scaffolds share a similar structure with mineral crystals dispersing homogeneously in the collagen fiber grid. The diameters of the mineral crystals were in the range of 150–200 *μ*m without significant differences between HTPS and HAPS, and HTPS was more porous than HAPS.

### 3.2. EDX Analysis

The EDX spectra were obtained for HAPS and HATS to determine the element composition. The EDX results (Table 2) show that both samples had similar elemental compositions, including carbon, oxygen, and nitrogen, which were derived from collagen and calcium and phosphorus were derived from the mineral components. HTPS had a lower calcium/phosphorus ratio than HAPS (HTPS: 1.58 : 1 and HAPS: 1.66 : 1), possibly because, during the preparation of HTP, HA reacted with H_3_PO_4_ and generated Ca_3_(PO_4_)_2_ in an acidic environment:

(1) $$\begin{array}{c} {\mathrm{Ca}}_{10}{\left({\mathrm{PO}}_{4}\right)}_{6}{\left(\mathrm{OH}\right)}_{2}+H^{+}⟶{\mathrm{Ca}}^{2+}+{{\mathrm{PO}}_{4}}^{3-}⟶{Ca}_{3}{\left(\;{PO}_{4}\;\right)}_{2} \end{array}$$

In the HAPS, the molar ratio of Ca/P accords with HA (1.67). In the HTPS, the molar ratio was less than 1.67. After calculation, the HA/*β*-TCP ratio in HTPS was found to be 1 : 1.

### 3.3. FTIR Analysis

**Figure 2:**
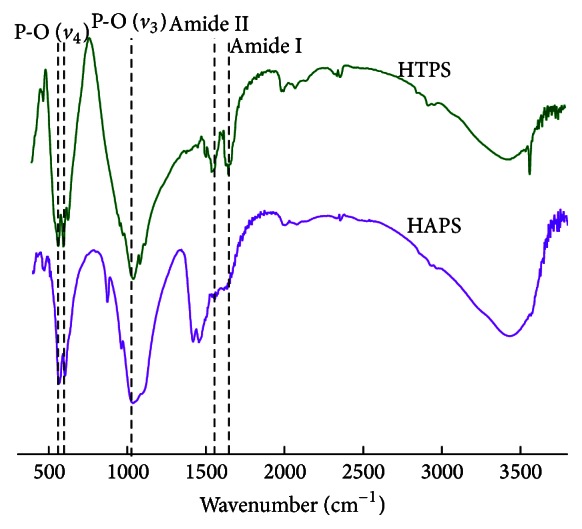
FTIR patterns of HTPS and HAPS. Both the groups share the same organic and inorganic composition. Note: green line: HTPS; purple line: HAPS. Dashed lines indicate similar symbolic peaks of the two groups: 1030 cm^−1^: *v*_3_ vibration of PO_4_^3−^; 602, 566 cm^−1^: *v*_4_ vibration of PO_4_^3−^; 1547 cm^−1^: Amide II; and 1600 cm^−1^: Amide I.

The FTIR spectra (Figure 2) of both groups show typical peaks at 1030 cm^−1^ and 602/566 cm^−1^, which are indicative of the *v*_3_ and *v*_4_ vibrations of PO_4_^3−^, and at 1547 and 1600 cm^−1^, which represent collagen (COO^−^). Such patterns are consistent with the collagen and mineral (HA or HA/TCP) components in each of the samples. However, the peaks at 1547 and 1600 cm^−1^ demonstrated different patterns in HTPS and HAPS. In HTPS, the peaks were sharper than those in HAPS because of the relatively low hydroxyl content, which could form hydrogen bonds with carbonyl and result in weakened vibrations of carbonyl. Therefore, the peaks of COO^−^ would become less sharp with higher hydroxyl content, and the addition of *β*-TCP, which lacks the hydroxyl group, would sharpen the characteristic peaks of COO^−^.

### 3.4. XRD Analysis

**Figure 3:**
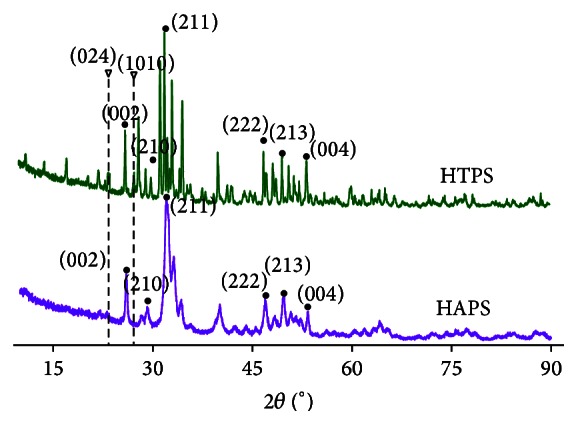
XRD spectra of HTPS and HAPS. Note: green line: HTPS; purple line: HAPS. Note: round symbols indicate the symbolic peaks of hydroxyapatite and triangles at 23.4°–23.7° and 26.9°–27.4° demonstrate the symbolic peaks of *β*-TCP in CHTS.

The XRD spectra (Figure 3) indicate significant peaks the RDin the 23.4°–23.7° and 26.9°–27.4° ranges for HTPS (International Center for Diffraction Data, Joint Committee on Powder Diffraction Standards, CPDS number 41-0487) and a much lower peak at 40.4°–40.9°, which indicates the TCP phase; HAPS does not have such features. The HA peaks of HTPS overlapped with those of HAPS due to a similar Ca_5_(PO_4_)_3_(OH) phase. The peaks between 25° and 35° in both the groups indicated the amorphous structure of collagen [36].

### 3.5. Mechanical Properties (Young's Modulus)

**Figure 4:**
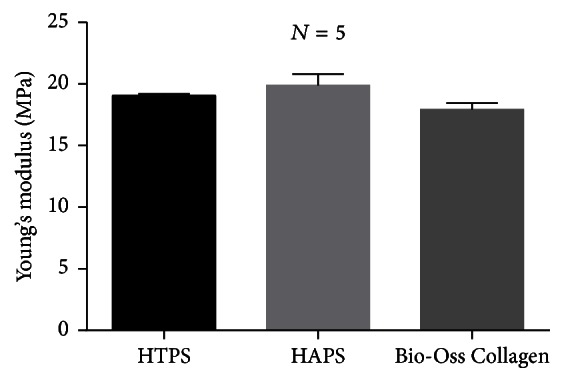
Young's moduli of HTPS and HAPS.

Figure 4 indicates that Young's modulus was 19.05 ± 0.088 (Mpa) in HTPS, 19.88 ± 0.530 (Mpa) in HAPS, and 17.93 ± 0.292 (Mpa) in Bio-Oss Collagen, respectively. No significant difference was observed between the three groups.

### 3.6. Porosity Analysis

**Figure 5:**
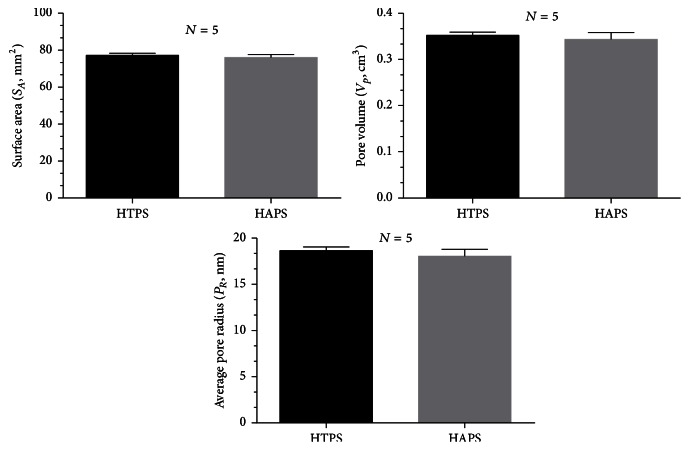
Porosity analysis of HTPS and HAPS.

The *S*_*A*_, *V*_*p*_, and *P*_*R*_ of HTPS and HAPS are shown in Figure 5. The *S*_*A*_ and *V*_*p*_ of both groups were similar. Although the values of *S*_*A*_, *V*_*p*_, and *P*_*R*_ of HAPS were less than those of HTPS, no significant differences were observed (HTPS: *S*_*A*_:  77.17 ± 0.68 mm^2^, *V*_*p*_:  0.3520 ± 0.004 cm^2^, and *P*_*R*_:  18.64 ± 0.23 nm; HAPS: *S*_*A*_:  76.03 ± 0.97 mm^2^, *V*_*p*_:  0.3433 ± 0.01 cm^2^, and *P*_*R*_:  18.04 ± 0.43 nm).

### 3.7. Water Absorption Behavior of HTPS and HAPS

**Figure 6:**
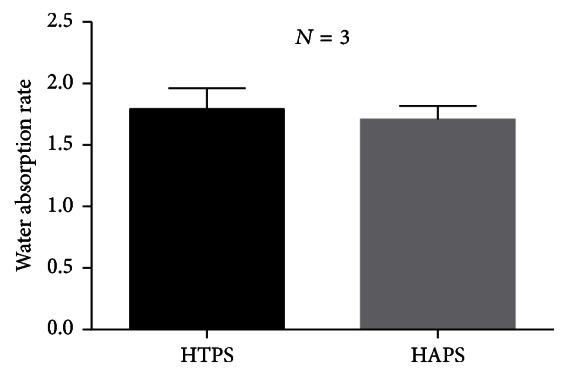
Water absorption rate of HTPS and HAPS.

The water absorption rate of HTPA and HAPS is shown in Figure 6. The absorption rate of HTPS was slightly lower than that of HAPS (HTPS: 1.793 ± 0.08410 and HAPS: 1.709 ± 0.05476), but the difference was not significant. This indicates that the water absorption ability and stability in aqua was similar for HAPS and HTPS. Such differences could be attributed to the higher porosity of HTPS, which would also enhance the exchange of substances in a cell growth environment.

### 3.8. MTT Assays

**Figure 7:**
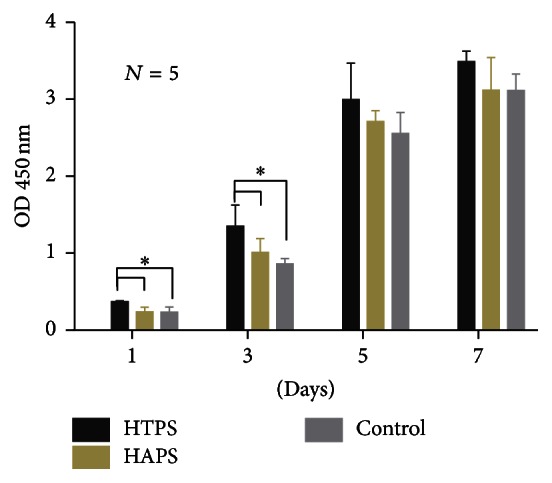
MTT line graph of HTPS and HAPS.

Cell proliferation on the scaffolds can reflect the toxicity and biocompatibility of the scaffold materials. MTT assays were, therefore, conducted to determine the proliferation of ADSCs on both HAPS and HATS. The growth curves (Figure 7) indicated that the overall growth tendencies in both groups were similar during the culture process. However, the cell proliferation was higher on HTPS from day 1 to day 7; particularly, significant differences could be detected on the first and third day. This is partly because the fast degradation of *β*-TCP led to an increase in the concentration of calcium and phosphate during this period. The growth tendency of the cells on HAPS and in the control group did not show significant differences at all the four indicated time points.

### 3.9. Gene Expression and Immunofluorescence Assay

**Figure 8:**
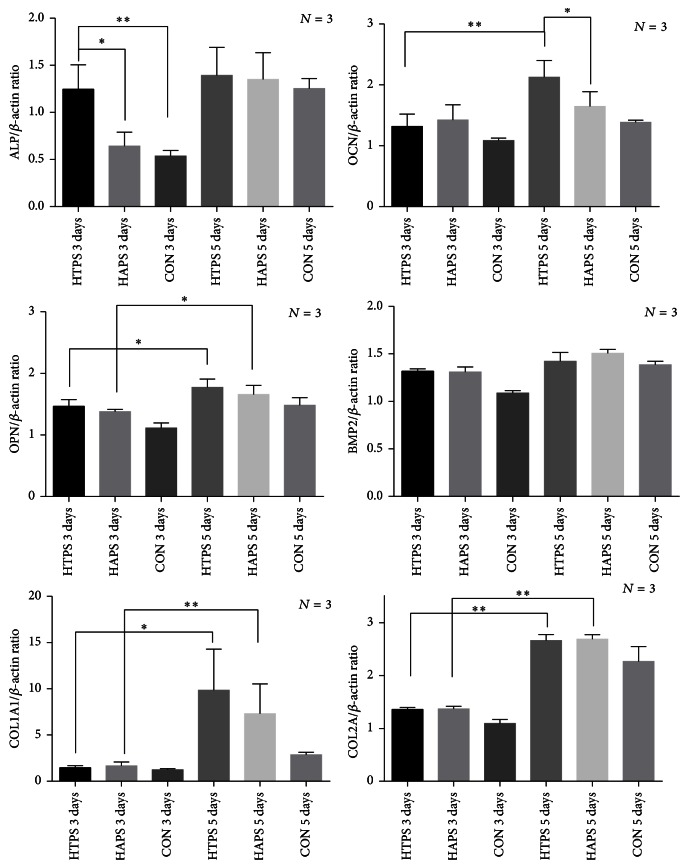
Gene expression of ALP, BMP-2, OCN, OPN, COL1A1, and COL2A in ADSCs cultured on HAPS and HTPS for 3 and 5 days. Note: ^*∗*^ *P* < 0.05, ^*∗∗*^ *P* < 0.01.

Osteogenesis-related gene expression could reflect the differentiation of ADSCs into osteoblasts, the key seed cells in bone tissue engineering. After the culture of ADSCs for 3 and 5 days, the mRNA levels of ALP, BMP-2, OCN, OPN, COL1A1, COL2A, and ACTIN were examined using RT-qPCR, and the results are shown in Figure 8. The expression of ALP in the cells cultured with HTPS on day 3 was significantly higher than that in the cells cultured with HAPS. On day 5, both groups showed similar ALP expression. OCN expression increased significantly from the third to the fifth day in both groups, and the cells cultured with HTPS showed a significant higher level than those cultured with HAPS. The OPN mRNA level in both groups increased significantly from the third to the fifth day, but the difference between the groups was not significant. Similarly, the expression of BMP2 increased slightly in both groups from the third to the fifth day, but no significant differences were observed between the groups. The expression of both COL1A1 and COL2A increased significantly from the third to the fifth day in both groups, but the level of COL1A1 was much higher than that of COL2A.

**Figure 9:**
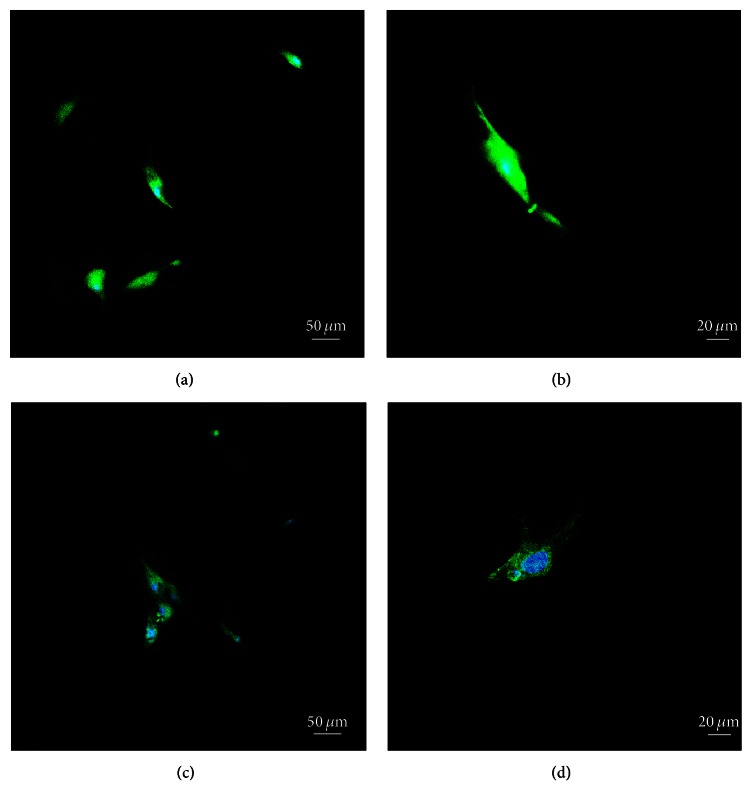
Fluorescence images of the ADSC seeded on HAPS and HTPS. Note: (a and b): ADSCs on HAPS at 24 h, 10x and 20x objective lenses, respectively, and (c and d): ADSCs on HTPS at 24 h, 10x and 20x objective lenses, respectively.

The cytoskeleton is a dynamic system of many cellular functions and could be an excellent indicator of the behavior of the ADSCs (Figure 9). By staining the cytoskeleton and nucleus, the morphology of the ADSCs could be easily detected with fluorescence microscopy. At 24 h, the cells on HAPS (Figures 9(a) and 9(b)) exhibited an elongated spindle shape, which is typical of MSCs. However, the cells on HTPS started to show typical osteoblast-like morphologies with a big nucleus and polygonal appearance (Figures 9(c) and 9(d)).

## 4. Discussion

Bioscaffolds have played an important role as one of three main elements of tissue engineering [47] and have been explored widely. In this study, type-I collagen composite scaffolds with two different mineral particles were prepared. Pure water was used to dissolve the collagen with mechanical stirring instead of an acid solution (e.g., acetic acid) to avoid the irritation caused by acidic solutions. To improve the mechanical property of collagen, crosslinking using glutaraldehyde [48] or EDC/NHS [49] was conducted. However, the inevitable residual would cause cytotoxicity and influence the healing process. Therefore, mechanical pressing was used to enhance the strength of the composite scaffolds without any negative effects on the biocompatibility.

Compared with HA prepared by chemical synthesis, inorganic bovine bone has better bone formation ability because its components and microstructure are similar to that of the natural bone [50–52]. In this study, we prepared two mineral particles, HAP and HTP. In the preparation of HTP, the addition of H_3_PO_4_ enabled a portion of HA to be transformed into TCP. As a result, the proportion of calcium and phosphorus in HTP was lower than that in HAP, as determined from the EDX spectrum (1.58 : 1 versus 1.66 : 1). XRD and FTIR analyses also confirmed the existence of *β*-TCP. HTP degrades and resolves faster because of the high biodegradation rate and solubility of *β*-TCP, which is suitable for tissue engineering applications [53]. With the addition of the *β*-TCP solution, the concentration of calcium and phosphate in the surroundings promotes osteogenesis by favoring the synthesis of osteoinductive growth factors and by upregulating adenosine signaling in phosphate metabolism [54–56]. The differentiation of osteoblasts and the subsequent bone formation, including the secretion and mineralization of ECM, can also be facilitated with the use of *β*-TCP [57]. Therefore, the use of *β*-TCP in HTPS could attain better biological effects than using HA alone.

Excellent mechanical property not only provides a mechanical support for bone formation, but also makes the materials conducive for use in clinical applications. Both HAPS and HTPS have Young's moduli similar to that of Bio-Oss Collagen, which has been widely used in clinical practice [58, 59]. Although we were unable to attain a Young modulus equivalent to that of the natural bone, the mechanical strength achieved by using these biomaterials is stronger than that obtained by using collagen alone [60] and is enough for dental applications in which the tensile stress is relatively lower [46]. Properties such as water absorption and porosity can allow the blood to infiltrate the graft quickly when implanted in the recipient and allow material exchange, which could promote cell proliferation. Besides, good water absorption can enable doctors to manipulate grafts conveniently by mixing the materials with normal saline or blood. No significant difference was detected in the water absorption rate between the two groups.

Stem cell-based therapy has become a promising tool in some fields such as craniofacial bone regeneration and spine surgery, and a randomized controlled clinical trial has shown positive effects of such therapy [61]. In our study, we tested the effects of the two types of biomaterial scaffolds on the proliferation and osteogenic differentiation capacity of ADSCs. Additionally, fluorescence images provided direct evidence indicating that the ADSCs cultured on HTPS could achieve osteogenesis earlier than those on HAPS. The MTT assays also suggest that the cells in both the groups showed higher (HTPS) or similar (HAPS) proliferation activity compared with the control group. However, from the first day, cell proliferation on HTPS was higher than that on HAPS, and this trend was observed until day 7. RT-PCR results indicate that the expression of all the osteoblastic genes except BMP2 was upregulated in both the groups. ALP is an extensively studied enzyme that is produced by osteoblasts, making it an important osteoblastic indicator. Osteocalcin (OCN) appears in the late stage of osteoblastic differentiation, while osteopontin (OPN) appears in the early stage [60]. In the HTPS group, ALP was upregulated significantly both on the third and on the fifth day. Both groups showed similar ALP expression on the fifth day. Similarly, both OCN and OPN were upregulated on the fifth day. The expression of these genes was in accordance with the results reported in previous literature on the osteogenic differentiation of mesenchymal cells on a TCP matrix [62–64]. COL1 and COL2 are two important components of the ECM. The expression of COL1 and COL2 increased in both the groups, indicating an increase in the synthesis of nonmineralized ECM, which is an important step in bone formation [60]. This phenomenon could be attributed to the function of collagen, whose ability to enhance collagen synthesis has been studied extensively [65]. Therefore, although both the scaffolds could enhance the osteogenic differentiation capacity of the ADSCs, HTPS could achieve a better result, which was also verified by the fluorescence images. The osteogenic differentiation also occurred earlier in the HTPS group than in the HAPS group.

## 5. Conclusion

In this study, two composite collagen scaffolds, HAPS and HTPS, were prepared using mineral particles distributed homogeneously in type-I collagen grids. HTPS contained 50%  *β*-TCP, which enabled better performance with regard to the promotion of the osteogenic differentiation of the ADSCs. Therefore, HTPS can be a novel candidate for use in stem cell-based therapy.

## Figures and Tables

**Table 1:** Primer sequences for RT-qPCR.

Primer number	Primer sequence (5′-3′)	Target gene	Product size (bp)
osgD145	CGACAGCAAGCCCAAGAG	ALP	110
osgD146	GTGGAGACGCCCATACCA		

osgD155	ACATCCGCTCCACAAACG	BMP-2	132
osgD156	GGTGCCACGATCCAGTCA		

osgD149	CTTCTCAGAGCCTCAGTCC	OCN	129
osgD150	ACCGTAGATGCGTTTGTAG		

ACTINF	ACTCGCTGCGCTCGGTCGTT	ACTIN	125
ACTINR	CCTTTTGCTGGCCTTTTGCTCAC		

osgD286	CACTCCAATCGTCCCTAC	OPN	127
osgD287	GTCCTCATCTGTGGCATC		

osgD316	CCCTGGACAGCCTGGACTT	COL1A1	95
osgD317	CATAGGACATCTGGGAAGCAA		

osgD290	ACCTTGGACGCCATGAAA	COL2A	102
osgD291	CTTGCTGCTCCACCAGTTT		

**Table 2:** Results of EDX analysis for HTPS and HAPS.

Element	At%	At%
	HTPS	HAPS
CK	39.64	48.49
NK	05.40	06.12
OK	34.17	28.74
NaK	00.69	00.56
MgK	00.44	00.30
PK	07.47	05.62
ClK	00.15	00.93
CaK	12.04	09.28
